# An RLP23/Cf‐9^TM‐IC^ Chimeric Receptor Enhances nlp24‐Triggered Immunity and Resistance to *Phytophthora nicotianae* in *Nicotiana benthamiana*

**DOI:** 10.1111/mpp.70307

**Published:** 2026-06-30

**Authors:** Xiaodie Huo, Siying Pan, Hongju Xiao, Ling Wu, Lichi Zhong, Qiang Cheng

**Affiliations:** ^1^ Key Laboratory of Forest Genetics and Biotechnology of Ministry of Education, Co‐Innovation Center for Sustainable Forestry in Southern China, Nanjing Forestry University Nanjing China

**Keywords:** Cf‐9, chimeric receptor, hypersensitive response, nlp24, NRC3, *Phytophthora nicotianae*

## Abstract

Receptor‐like protein AtRLP23 from *Arabidopsis thaliana* perceives the conserved microbial peptide nlp24 derived from necrosis‐ and ethylene‐inducing peptide 1‐like proteins (NLPs), but immune outputs are often weak in heterologous systems. Here, we engineered RLP23/Cf‐9 chimeric receptors by fusing the extracellular domain of AtRLP23 to either the Cf‐9 intracellular region (IC) or the Cf‐9 transmembrane–intracellular region (TM–IC), and assessed their activity in *Nicotiana benthamiana*. In transient expression assays, AtRLP23 alone did not trigger hypersensitive cell death after infiltration with 10 μM nlp24, whereas both chimeras conferred nlp24‐induced cell death, with RLP23/Cf‐9^TM‐IC^ producing markedly stronger macroscopic symptoms than RLP23/Cf‐9^IC^. Stable transgenic *N. benthamiana* expressing an untagged RLP23/Cf‐9^TM‐IC^ chimera showed enhanced nlp24 responsiveness, including stronger seedling growth inhibition and cell death after repeated 10 μM nlp24 treatment. In these plants, virus‐induced gene silencing showed that chimera‐mediated cell death requires SOBIR1 and the helper NLR NRC3, and transient *NRC3* expression further sensitized the response, enabling cell death after a single 0.1 μM nlp24 treatment. In addition, the chimera significantly enhanced resistance to the oomycete *Phytophthora nicotianae*. Together, these results identify RLP23/Cf‐9^TM‐IC^ as an effective chimeric receptor and reveal transient *NRC3* expression as a practical step to further potentiate nlp24‐triggered output in *N. benthamiana*.

## Graphical abstract

We engineered an RLP23/Cf‐9^TM‐IC^ chimeric receptor that confers robust nlp24‐triggered immunity in *Nicotiana benthamiana*. Notably, this enhanced cell‐death output strictly depends on the helper NLR NRC3. This engineered module provides a powerful strategy to improve resistance against the destructive oomycete pathogen *Phytophthora nicotianae*.

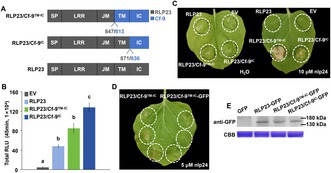

The plant innate immune system relies on cell‐surface pattern recognition receptors (PRRs) to detect pathogen‐associated molecular patterns (PAMPs) and activate pattern‐triggered immunity (PTI). These receptors are generally classified as receptor‐like kinases (RLKs) or receptor‐like proteins (RLPs; Macho and Zipfel 2014). Because RLPs lack an intracellular kinase domain, they typically signal through constitutive association with SOBIR1 and ligand‐induced recruitment of the co‐receptor BAK1 (Liebrand et al. 2014). Beyond this core RLP signaling module, some RLP‐mediated outputs, particularly hypersensitive cell death, can also depend on helper NLRs. In *Nicotiana benthamiana*, studies with tomato Cf receptor‐like proteins, including Cf‐4 and Cf‐9, have identified NRC3 as the principal helper NLR mediating cell‐surface receptor‐triggered hypersensitive cell death (Kourelis et al. 2022).

Necrosis‐ and ethylene‐inducing peptide 1‐like proteins (NLPs) are broadly distributed among bacteria, fungi, and oomycetes (Seidl and Van Den Ackerveken 2019). For example, the devastating solanaceous pathogen *Phytophthora nicotianae* secretes multiple NLPs during infection (Panabières et al. 2016). In *Arabidopsis thaliana* , AtRLP23 recognizes conserved NLP‐derived peptides such as nlp20 and nlp24 and activates PTI through SOBIR1‐ and BAK1‐dependent receptor complexes (Albert et al. 2015). Transfer of AtRLP23 into heterologous species can confer NLP responsiveness and improve disease resistance, but immune outputs are often limited and do not necessarily culminate in robust hypersensitive cell death (Albert et al. 2015; Zhao and Cheng 2023).

Cf family receptors are known for their strong cell‐death output, making Cf‐9‐derived C‐terminal modules attractive tools for chimeric receptor engineering in Solanaceae. Previous studies showed that Cf‐9 signalling modules can strengthen engineered PRR outputs in tobacco and tomato (Wu et al. 2019; Yang et al. 2025). Here, we investigated whether distinct Cf‐9 C‐terminal modules differ in their ability to strengthen RLP23‐mediated nlp24 responses in *N. benthamiana*, and whether chimera‐mediated output could be further potentiated through downstream signalling components. We further tested whether the most effective chimera improved resistance to *P. nicotianae* .

To test whether Cf‐9‐derived C‐terminal modules could enhance RLP23‐mediated immune outputs, we generated two chimeric receptors (Figure 1A). In RLP23/Cf‐9^IC^, the ectodomain and transmembrane (TM) domain of AtRLP23 were fused to the intracellular (IC) region of Cf‐9. In RLP23/Cf‐9^TM‐IC^, the ectodomain of AtRLP23 was fused to the Cf‐9 TM‐IC region. Following transient expression in *N. benthamiana*, nlp24 treatment induced robust reactive oxygen species (ROS) production in leaves expressing RLP23/Cf‐9^TM‐IC^, RLP23/Cf‐9^IC^ or RLP23, whereas the empty‐vector control showed only a low background signal (Figure 1B; full ROS kinetic curves are shown in Figure S1). Quantitative analysis indicated that both chimeric receptors retained nlp24 responsiveness at the level of early PTI outputs, with ROS accumulation comparable to or slightly higher than that of RLP23. In contrast, the cell‐death phenotype differed markedly among the constructs. Upon treatment with 10 μM nlp24 at 48 h after agroinfiltration, RLP23/Cf‐9^TM‐IC^ consistently induced strong macroscopic cell death in all independent assays (10/10 leaves), whereas RLP23/Cf‐9^IC^ produced weaker and less uniform necrosis, typically appearing as patchy lesions (8/10 leaves). No visible cell death was observed in leaves expressing RLP23 under the same conditions (Figures 1C and S2A). We further examined a C‐terminal GFP fusion of RLP23/Cf‐9^TM‐IC^ and found that GFP tagging attenuated cell‐death induction and reduced reproducibility relative to the corresponding untagged construct (Figures 1D and S2B). In addition, immunoblot analysis of GFP‐tagged RLP23, RLP23/Cf‐9^TM‐IC^, and RLP23/Cf‐9^IC^ showed comparable protein accumulation among the three constructs (Figures 1E and S9). Together, these results indicate that the Cf‐9 TM‐IC module is sufficient to confer a clear and reproducible nlp24‐triggered cell‐death output, whereas the Cf‐9 IC module alone is less effective.

**FIGURE 1:**
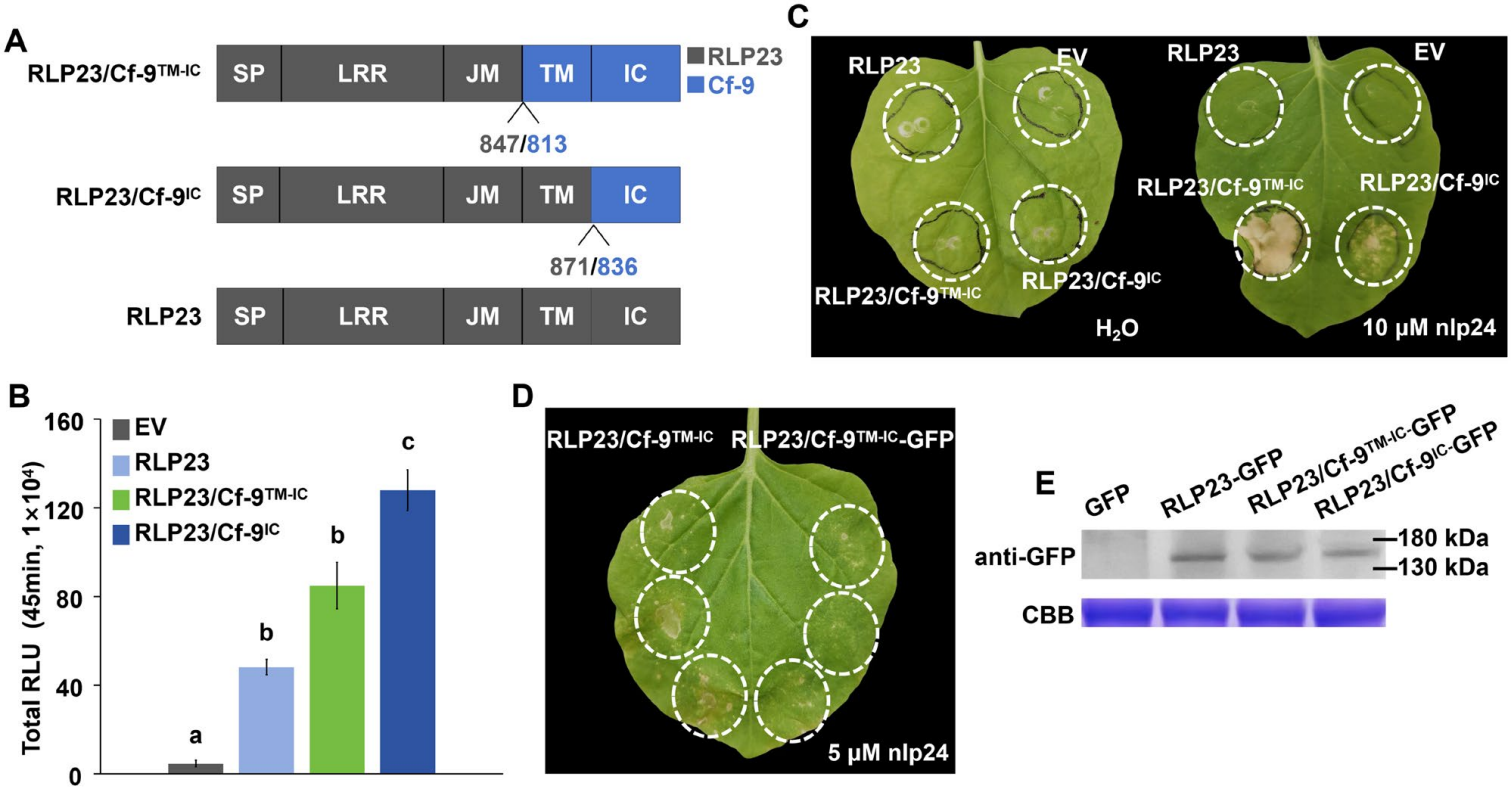
The Cf‐9^TM‐IC^ module enhances nlp24‐triggered cell‐death output without compromising early reactive oxygen species (ROS) responses in *Nicotiana benthamiana*. (A) Schematic representation of RLP23 and the chimeric receptors RLP23/Cf‐9^TM‐IC^ and RLP23/Cf‐9^IC^. Regions derived from RLP23 and Cf‐9 are shown in grey and blue, respectively. Numbers above the junctions indicate the exact fusion points, with grey numbers denoting amino acid positions in RLP23 and blue numbers denoting amino acid positions in Cf‐9. SP, signal peptide; LRR, leucine‐rich repeat; JM, juxtamembrane region; TM, transmembrane region; IC, intracellular region. (B) ROS production in leaf discs from *N. benthamiana* transiently expressing RLP23, RLP23/Cf‐9^TM‐IC^, RLP23/Cf‐9^IC^ or the empty vector (EV) after treatment with nlp24 (1 μM). Total ROS accumulation over 45 min is shown. Data are means ± SEM from three independent experiments. Different letters indicate significant differences among groups (*p* < 0.05, one‐way ANOVA followed by Tukey's multiple comparison test). (C) Macroscopic cell‐death phenotypes induced by the indicated constructs in *N. benthamiana* leaves. At 48 h after agroinfiltration, infiltration sites were treated with water (left) or nlp24 (10 μM; right), and photographs were taken 4 days later. Dashed circles indicate infiltration sites. (D) Effect of C‐terminal GFP tagging on RLP23/Cf‐9^TM‐IC^‐mediated cell death. Leaves expressing untagged or C‐terminally GFP‐tagged RLP23/Cf‐9^TM‐IC^ were treated with nlp24 (5 μM), and photographs were taken 4 days later. Dashed circles indicate infiltration sites. (E) Immunoblot analysis of C‐terminally GFP‐tagged RLP23, RLP23/Cf‐9^TM‐IC^, and RLP23/Cf‐9^IC^ expressed in *N. benthamiana* leaves. Total proteins were extracted at 48 h after agroinfiltration and detected with an anti‐GFP antibody. Coomassie Brilliant Blue staining is shown as a loading control. All experimental details of this study are described in Method S1, and primer sequences used in this study are provided in Table S1.

To validate the activity of the engineered receptor in a stable genetic background, we generated transgenic *N. benthamiana* lines expressing either RLP23 or RLP23/Cf‐9^TM‐IC^. Two independent lines for each construct were selected and advanced to the T_2_ generation for analysis. Reverse transcription‐quantitative PCR (RT‐qPCR) confirmed substantial accumulation of the corresponding transgene transcripts in all four lines, with neither of the two RLP23/Cf‐9^TM‐IC^ lines showing higher transcript levels than the RLP23 lines (Figure 2A). Under normal growth conditions, these transgenic plants showed no obvious developmental abnormalities compared with wild‐type (WT) plants, indicating that transgene expression did not cause constitutive growth defects in the absence of elicitor treatment (Figure S3A).

**FIGURE 2:**
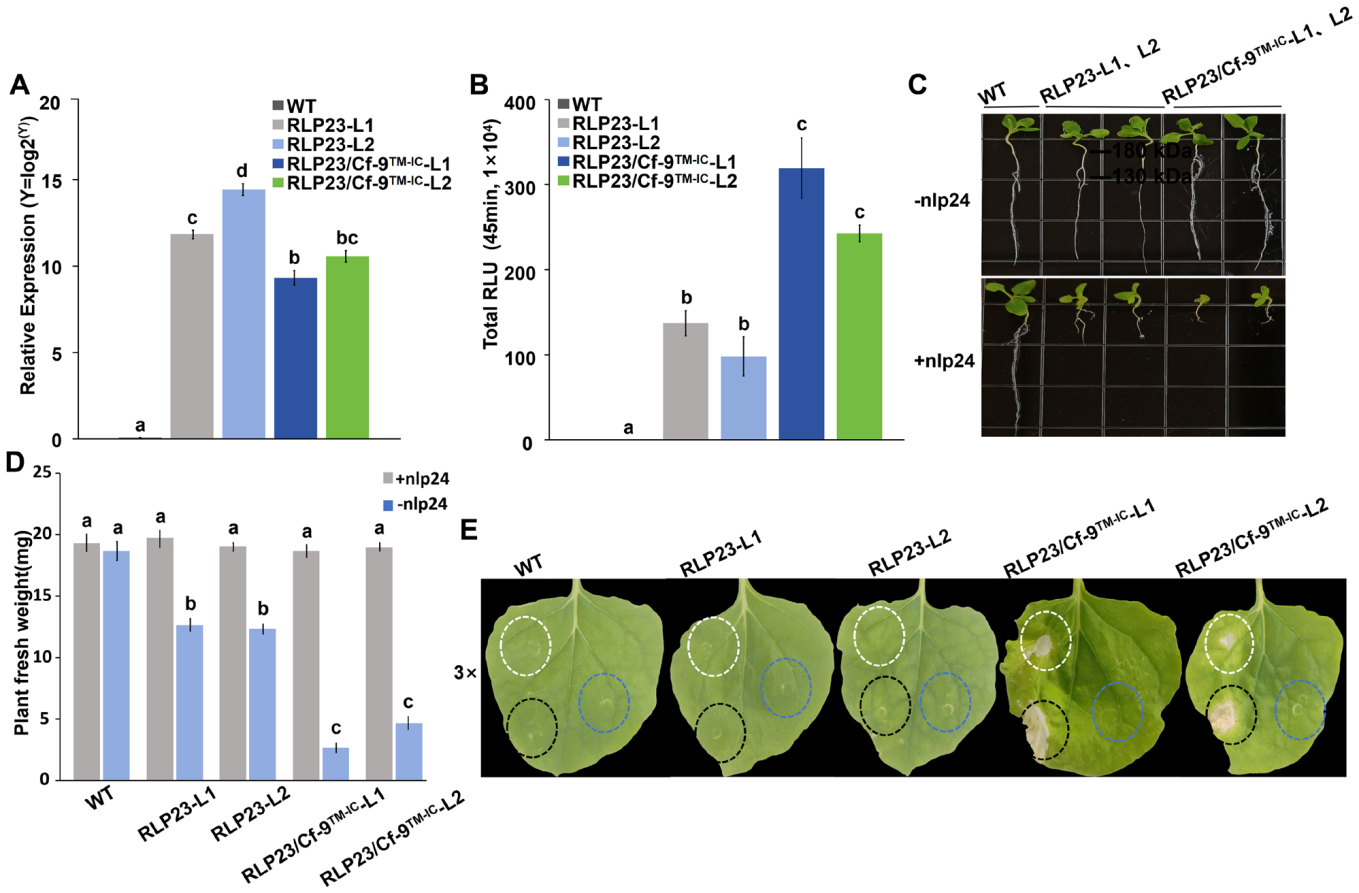
Stable expression of RLP23/Cf‐9^TM‐IC^ confers stronger downstream nlp24 responses than RLP23 in *Nicotiana benthamiana*. (A) Relative transcript levels of the transgenes in two independent RLP23 lines (RLP23‐L1 and RLP23‐L2) and two independent RLP23/Cf‐9^TM‐IC^ lines (RLP23/Cf‐9^TM‐IC^‐L1 and RLP23/Cf‐9^TM‐IC^‐L2). Expression values were calculated using the 2^−ΔΔ*C*t^ method with *NbEF1α* as the internal reference and are presented as log_2_‐transformed values [*y* = log_2_(2^−ΔΔ*C*t^)]. Data are means ± SEM from three biological replicates. (B) Reactive oxygen species (ROS) production in leaf discs from wild‐type (WT) and transgenic plants after treatment with nlp24 (1 μM). Total ROS accumulation over 45 min is shown. Data are means ± SEM from three independent experiments. (C) Seedling growth inhibition assay. Seedlings were germinated on solid Murashige and Skoog (MS) medium and then incubated in liquid MS medium in the absence (−nlp24) or presence (+nlp24) of 10 μM nlp24 for 7 days. Representative photographs are shown. (D) Fresh weight of seedlings shown in (C) (*n* = 9). (E) Cell‐death phenotypes in leaves of WT and transgenic plants after repeated treatment with nlp24. The same leaf areas were treated three times (3×) with 5 μM nlp24 (white dashed circles), 10 μM nlp24 (black dashed circles), or water (blue dashed circles), with each subsequent infiltration performed after the previous infiltrated solution had dried. Photographs were taken 7 days after the final treatment. In (A), (B), and (D), different letters indicate statistically significant differences among groups (*p* < 0.05, one‐way ANOVA followed by Tukey's multiple comparison test).

Upon treatment with 1 μM nlp24, all four transgenic lines exhibited markedly stronger ROS production than WT plants (Figure 2B, full ROS kinetic curves are shown in Figure S4), and ROS production was significantly higher in the RLP23/Cf‐9^TM‐IC^ lines than in the RLP23 lines. The different ROS patterns between the transient and stable assays may reflect differences in experimental context, with a clearer increase in RLP23/Cf‐9^TM‐IC^‐mediated ROS output observed in the stable transgenic lines. We next examined seedling growth responses in liquid Murashige and Skoog (MS) medium supplemented with 10 μM nlp24. Compared with WT, both RLP23 and RLP23/Cf‐9^TM‐IC^ lines showed clear growth inhibition and significantly reduced fresh weight (Figures 2C,D and S3B). Notably, fresh weight reduction was significantly greater in the RLP23/Cf‐9^TM‐IC^ lines than in the RLP23 lines, indicating that the chimera strengthens the downstream consequences of nlp24 perception in planta.

We then assessed macroscopic cell‐death output in the stable lines. Compared with the transient expression assays of RLP23/Cf‐9^TM‐IC^, nlp24‐induced cell death in the stable transgenic lines was weaker and more condition‐dependent. A single treatment with 5 μM or 10 μM nlp24 induced only weak and infrequent visible cell death in RLP23/Cf‐9^TM‐IC^ plants (Figure S3C). However, when the same leaf area was treated three times with 10 μM nlp24 after each infiltrated solution had dried, a procedure intended to increase local peptide concentration in the apoplast, reproducible macroscopic cell death was observed in both RLP23/Cf‐9^TM‐IC^ lines, whereas no visible cell death was detected in the RLP23 lines under the same regime (Figure 2E). Together, these results show that stable expression of RLP23/Cf‐9^TM‐IC^ confers stronger downstream nlp24 responses than RLP23, most clearly in seedling growth inhibition and reproducible cell‐death output in *N. benthamiana*.

To test whether RLP23/Cf‐9^TM‐IC^‐mediated cell death depends on SOBIR1 and NRC3, we used tobacco rattle virus (TRV)‐based virus‐induced gene silencing (VIGS) in WT, RLP23 transgenic, and RLP23/Cf‐9^TM‐IC^ transgenic *N. benthamiana* plants. In the TRV:EV (empty vector) background, treatment with 10 μM nlp24 consistently induced clear necrotic symptoms in the RLP23/Cf‐9^TM‐IC^ lines, whereas no obvious cell death was observed in WT or RLP23 lines. By contrast, nlp24‐triggered necrosis in the chimera lines was abolished in both TRV:SOBIR1‐ and TRV:NRC3‐treated plants. Transient expression of *BAX* induced strong necrosis in all silencing backgrounds, indicating that the general capacity for cell death remained intact (Figures 3A and S5A). Silencing of *NRC3* was confirmed by RT‐qPCR (Figure 3B), whereas the efficiency of *SOBIR1* silencing was verified in representative genotypes (Figure S5B). We further examined whether *NRC3* silencing affected the early ROS response to nlp24 in WT plants and representative transgenic lines, RLP23 L1 and RLP23/Cf‐9^TM‐IC^ L1 (Figure S6). Compared with the corresponding TRV:EV controls, TRV:NRC3 treatment did not markedly reduce nlp24‐induced ROS production in either transgenic line.

**FIGURE 3:**
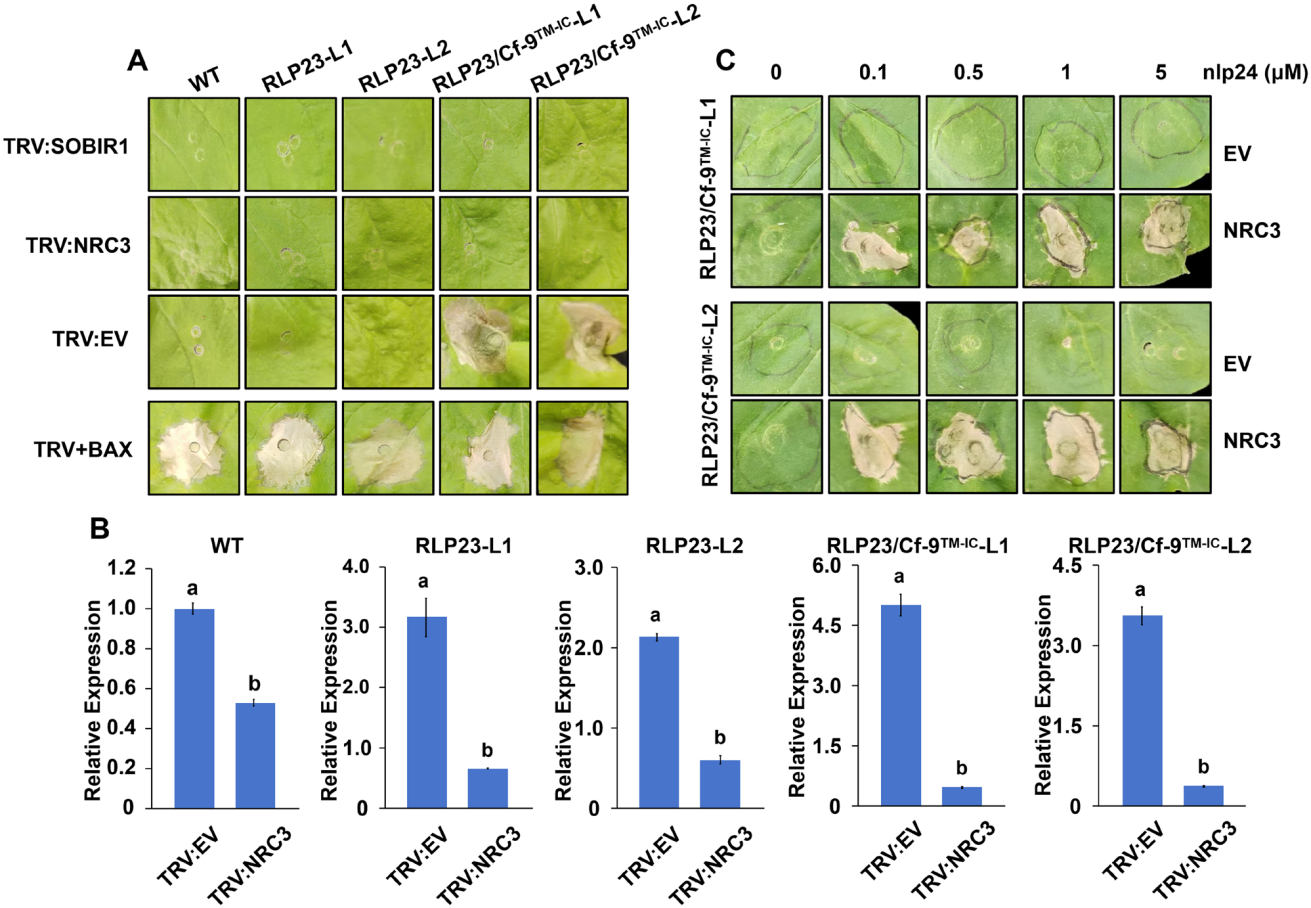
Effects of *SOBIR1* and *NRC3* silencing and transient *NRC3* expression on RLP23/Cf‐9^TM‐IC^‐mediated cell death. (A) Cell‐death phenotypes in wild‐type (WT), RLP23 transgenic lines (L1 and L2), and RLP23/Cf‐9^TM‐IC^ transgenic lines (L1 and L2) after tobacco rattle virus (TRV)‐based virus‐induced gene silencing (VIGS) of *SOBIR1* or *NRC3*. Plants carrying TRV:EV (empty vector) were used as controls. Leaves were treated three times (3×) with 10 μM nlp24 after each infiltrated solution had dried. BAX was used as a positive control for cell death. Photographs were taken 5 days after treatment. (B) Relative *NRC3* transcript levels in WT, RLP23, and RLP23/Cf‐9^TM‐IC^ plants after TRV‐based silencing. Data are means ± SEM from three biological replicates. Different letters indicate statistically significant differences between the TRV:EV control and TRV:NRC3 treatment within each indicated genotype (*p* < 0.05, Welch's unpaired *t *test). (C) Cell‐death phenotypes of RLP23/Cf‐9^TM‐IC^ transgenic lines (L1 and L2) after transient expression of *NRC3* or EV. Leaves were infiltrated with NRC3 or EV and then treated once with 0 μM (water), 0.1 μM, 0.5 μM, 1 μM, or 5 μM nlp24 at 48 h after agroinfiltration. Photographs were taken 5 days after treatment.

Given the requirement for NRC3, we next tested whether *NRC3* expression could further sensitize RLP23/Cf‐9^TM‐IC^ transgenic plants to nlp24. Transient expression of *NRC3* in RLP23/Cf‐9^TM‐IC^ transgenic plants markedly enhanced responsiveness, enabling clear macroscopic cell death at nlp24 concentrations as low as 0.1 μM, whereas the EV control produced only weak or restricted symptoms even at 5 μM (Figures 3C and S7). These results indicate that RLP23/Cf‐9^TM‐IC^‐mediated cell death requires both SOBIR1 and NRC3, and further show that transient *NRC3* expression acts as an effective potentiation step that lowers the threshold for nlp24‐triggered macroscopic cell death. Although *NRC3* silencing abolished nlp24‐triggered cell death in RLP23/Cf‐9^TM‐IC^ plants and transient NRC3 expression lowered the threshold for cell‐death induction, possible contributions from other NRC helpers cannot be excluded given the sequence similarity and functional redundancy within the NRC family.

We amplified and Sanger‐sequenced the full‐length CDS of an *NPP1* homologue from cDNA prepared from leaves infected with our laboratory‐maintained *P. nicotianae* isolate. The transcript encoded an nlp24 region identical to the synthetic nlp24 peptide used in our immune assays. On this basis, we next tested whether the RLP23/Cf‐9^TM‐IC^ chimera enhanced resistance to this isolate. Detached leaves from wild‐type (WT) plants, two RLP23 transgenic lines (L1 and L2), and two RLP23/Cf‐9^TM‐IC^ transgenic lines (L1 and L2) were inoculated with the pathogen and disease development was monitored. At 3 days post‐inoculation (dpi), WT leaves developed extensive water‐soaked lesions, whereas lesion development was reduced in all transgenic lines (Figures 4A and S8). This reduction was more pronounced in the RLP23/Cf‐9^TM‐IC^ lines, which retained more green tissue and showed more restricted lesion expansion than the RLP23 lines.

**FIGURE 4:**
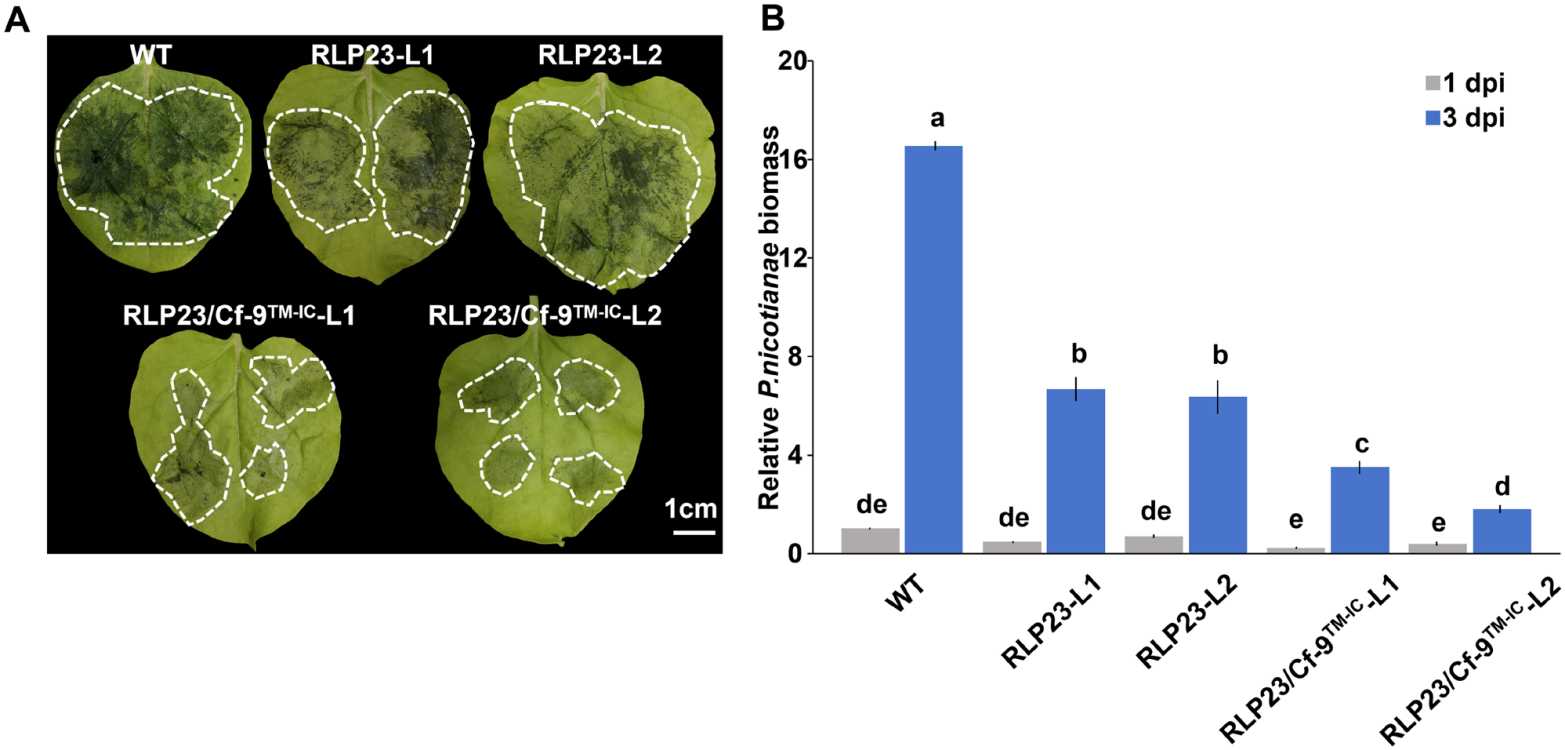
RLP23/Cf‐9^TM‐IC^ confers stronger resistance to *Phytophthora nicotianae* than RLP23 in *Nicotiana benthamiana*. (A) Representative detached‐leaf infection phenotypes of wild‐type (WT), RLP23 transgenic lines (L1 and L2), and RLP23/Cf‐9^TM‐IC^ transgenic lines (L1 and L2) at 3 days post‐inoculation (dpi) with *P. nicotianae* . Four inoculation sites were applied per detached leaf. Dashed lines indicate lesion boundaries. (B) Relative *P. nicotianae* biomass in infected leaves at 1 and 3 dpi, determined by quantitative PCR using *P. nicotianae* *β‐tubulin* as the pathogen marker and *NbEF1α* as the internal reference. One 2 cm leaf disc was collected around each inoculation site, and the four discs from the same leaf were pooled as one sample. Data are presented as means ± SEM of three biological replicates. Different letters indicate significant differences among genotypes at the same time point (*p* < 0.05, one‐way ANOVA followed by Tukey's multiple comparison test).

Quantification of pathogen biomass further supported these observations (Figure 4B). At 1 dpi, *P. nicotianae* biomass remained low in all genotypes and showed only minor differences among lines. By 3 dpi, biomass had increased markedly in WT leaves but remained significantly lower in all transgenic lines. Notably, both RLP23/Cf‐9^TM‐IC^ lines accumulated less pathogen biomass than the RLP23 lines, with the two chimera lines showing the strongest overall reduction. Together, these results indicate that stable expression of RLP23/Cf‐9^TM‐IC^ enhances resistance to *P. nicotianae* in *N. benthamiana* and provides stronger protection than RLP23 alone.

In summary, we generated RLP23/Cf‐9 chimeric receptors and evaluated their activities in *N. benthamiana*, and found that incorporation of the Cf‐9 TM‐IC region markedly enhanced nlp24‐triggered immune output compared with native RLP23. This enhancement was most evident in hypersensitive cell death and seedling growth inhibition. We further demonstrated that RLP23/Cf‐9^TM‐IC^‐mediated cell death depends on SOBIR1 and NRC3, and that transient expression of *NRC3* could further sensitize the chimera response, lowering the threshold for visible cell death. Across native RLP23 and the two chimeric receptor configurations, the magnitude of early ROS accumulation did not closely parallel the strength of macroscopic cell‐death output. Moreover, RLP23/Cf‐9^TM‐IC^‐mediated cell death was dependent on NRC3, whereas *NRC3* silencing did not impair nlp24‐triggered ROS production in the transgenic lines. This is consistent with previous work showing that NRC2/3/4 are not essential for flg22‐induced ROS burst and MAPK activation in *N. benthamiana* (Wu et al. 2020). Together, these observations are consistent with a model in which native RLP23, RLP23/Cf‐9^IC^, and RLP23/Cf‐9^TM‐IC^ can all support canonical early PTI outputs, whereas the Cf‐9 TM‐IC module may more effectively couple extracellular ligand perception to a functionally separable, NRC3‐associated cell‐death branch. In addition, stable expression of RLP23/Cf‐9^TM‐IC^ significantly improved resistance to *P. nicotianae* . This study identifies an effective Cf‐9‐derived signalling module for RLP23 engineering and shows that downstream signalling compatibility, especially NRC3, can be used to further potentiate output. Our results support the utility of Cf‐9‐derived signalling modules for improving engineered PRR output in solanaceous plants and suggest that optimization of downstream signalling components may provide an effective route to further enhance their performance.

## Author Contributions

**Siying Pan:** validation, formal analysis, investigation. **Lichi Zhong:** validation. **Qiang Cheng:** conceptualization, methodology, writing – original draft, funding acquisition, writing – review and editing, resources, project administration, supervision, validation, data curation. **Xiaodie Huo:** methodology, validation, visualization, writing – review and editing, writing – original draft, investigation, software, data curation, conceptualization, formal analysis. **Hongju Xiao:** investigation, validation. **Ling Wu:** conceptualization.

## Funding

This work was supported by the National Natural Science Foundation of China, 32572059.

## Conflicts of Interest

The authors declare no conflicts of interest.

## Supporting information


**Methods S1.** Experimental procedures.

**Table S1:** Primers used in this study.

**Figure S1:** Full reactive oxygen species (ROS) burst kinetics corresponding to Figure 1B in transient expression assays. ROS production in leaf discs from *Nicotiana benthamiana* transiently expressing the empty vector (EV), RLP23, RLP23/Cf‐9^TM‐IC^, or RLP23/Cf‐9^IC^ after treatment with 1 μM nlp24. Relative light units (RLU) were recorded over 45 min. The three panels represent three independent biological replicates; within each replicate, six leaf discs were used per treatment. Values are shown as means ± SEM.

**Figure S2:** Supplementary cell‐death phenotypes mediated by RLP23/Cf‐9 chimeric receptors in *Nicotiana benthamiana*. (A) Cell‐death phenotypes induced by the indicated constructs after treatment with H_2_O or 10 μM nlp24. At 48 h after agroinfiltration, infiltration sites were treated with H_2_O or nlp24, and photographs were taken 4 days later. The numbers on the right indicate the number of leaves showing macroscopic cell death out of the total number of leaves tested. Leaves were scored as positive when visible dry cell‐death symptoms were observed within the nlp24‐treated infiltration site, including patchy dry areas outside the injection wound; weak chlorosis alone or no visible cell‐death symptoms was not counted as positive. (B) Effect of C‐terminal GFP tagging on RLP23/Cf‐9^TM‐IC^‐mediated cell death. Leaves expressing untagged RLP23/Cf‐9^TM‐IC^ or C‐terminally GFP‐tagged RLP23/Cf‐9^TM‐IC^ were treated with 5 μM nlp24 at 48 h after agroinfiltration, and photographs were taken 4 days later.

**Figure S3:** Additional characterization of stable transgenic *Nicotiana benthamiana* lines expressing RLP23 or RLP23/Cf‐9^TM‐IC^. (A) Representative phenotypes of wild‐type (WT), RLP23 transgenic lines (L1 and L2), and RLP23/Cf‐9^TM‐IC^ transgenic lines (L1 and L2) grown under normal conditions. (B) Seedling growth inhibition assay of WT, RLP23, and RLP23/Cf‐9^TM‐IC^ lines in the absence (−nlp24) or presence (+nlp24) of 10 μM nlp24. Seedlings were germinated on solid Murashige and Skoog (MS) medium and then incubated in liquid MS medium with or without nlp24 for 7 days. Representative photographs are shown. (C) Cell‐death phenotypes in stable RLP23/Cf‐9^TM‐IC^ transgenic lines after single (1×) or repeated (3×) treatment with nlp24. Leaves were treated once or three times with 5 μM nlp24 (orange dashed circles), 10 μM nlp24 (black dashed circles), or H_2_O (blue dashed circles), and photographs were taken 7 days after the final treatment.

**Figure S4:** Full reactive oxygen species (ROS) burst kinetics corresponding to Figure 2B in stable transgenic lines. ROS production in leaf discs from wild‐type (WT), RLP23 transgenic lines, and RLP23/Cf‐9^TM‐IC^ transgenic lines after treatment with 1 μM nlp24. Relative light units (RLU) were recorded over 45 min. The three panels represent three independent biological replicates; within each replicate, six leaf discs were used per treatment. Values are shown as means ± SEM.

**Figure S5:** Additional characterization of SOBIR1 and NRC3 involvement in RLP23/Cf‐9^TM‐IC^‐mediated cell death. (A) Full‐leaf images corresponding to Figure 3A. Leaves of wild‐type (WT), RLP23 transgenic lines (L1 and L2), and RLP23/Cf‐9^TM‐IC^ transgenic lines (L1 and L2) were subjected to tobacco rattle virus (TRV)‐based virus‐induced gene silencing (VIGS) using TRV:SOBIR1, TRV:NRC3, or TRV:EV (empty vector), and then treated with 10 μM nlp24. BAX was used as a positive control for cell death. Photographs were taken 5 days after treatment. (B) Relative *SOBIR1* transcript levels in WT, RLP23, and RLP23/Cf‐9^TM‐IC^ plants after TRV‐based silencing. Data are means ± SEM from three biological replicates. Different letters indicate statistically significant differences between the TRV:EV control and TRV:SOBIR1 treatment within each indicated genotype (*p* < 0.05, Welch's unpaired *t*‐test).

**Figure S6:** *NRC3* silencing does not markedly reduce nlp24‐triggered reactive oxygen species (ROS) production in representative transgenic lines. (A) Quantification of ROS production in leaf discs from wild‐type (WT), representative RLP23 transgenic plants, and representative RLP23/Cf‐9^TM‐IC^ transgenic plants after treatment with 1 μM nlp24. Plants were subjected to toboacco rattle virus (TRV)‐based silencing using TRV:NRC3, with TRV:EV as the empty‐vector control. Relative light units (RLU) were recorded over 45 min. Data are shown as means ± SEM from three independent biological replicates, with six leaf discs per treatment in each replicate. Different letters indicate statistically significant differences among groups (*p* < 0.05, one‐way ANOVA followed by Tukey's multiple comparison test). (B) Full ROS burst kinetics corresponding to Figure S6A. The three panels represent three independent biological replicates; within each replicate, six leaf discs were used per treatment. Values are shown as means ± SEM. (C) Functional confirmation of *NRC3* silencing in the transgenic plants used for the ROS assay. Leaves were treated three times with 10 μM nlp24, and reduced cell‐death output in TRV:NRC3‐treated RLP23/Cf‐9^TM‐IC^ plants compared with the TRV:EV control confirmed effective suppression of the NRC3‐dependent cell‐death response. Photographs were taken 5 days after treatment.

**Figure S7:** Biological replicates showing NRC3‐mediated sensitization of RLP23/Cf‐9^TM‐IC^ transgenic plants to nlp24‐triggered cell death. Full‐leaf images and biological replicates corresponding to Figure 3C. Leaves of stable RLP23/Cf‐9^TM‐IC^ transgenic lines (L1 and L2) were transiently infiltrated with NRC3 or empty vector (EV) and then treated with H_2_O, 0.1 μM nlp24, 0.5 μM nlp24, 1 μM nlp24, or 5 μM nlp24. Photographs were taken 5 days after treatment.

**Figure S8:** Additional representative phenotypes and lesion area quantification of *Phytophthora nicotianae* infection on detached leaves. (A) Additional representative detached‐leaf infection phenotypes of wild‐type (WT), RLP23 transgenic lines, and RLP23/Cf‐9^TM‐IC^ transgenic lines at 3 days post‐inoculation (dpi) with *P. nicotianae* . (B) Quantification of lesion areas in detached leaves at 3 dpi. Lesion areas were measured from photographed leaves using ImageJ software. Data are presented as means ± SEM. Different letters indicate significant differences among genotypes (*p* < 0.05, one‐way ANOVA followed by Tukey's multiple comparison test).

**Figure S9:** Original uncropped images for Figure 1E.

## Data Availability

The data that support the findings of this study are available from the corresponding author upon reasonable request.
