# Supplementary material for: An RLP23/Cf‐9TM‐IC Chimeric Receptor Enhances nlp24‐Triggered Immunity and Resistance to Phytophthora nicotianae in Nicotiana benthamiana
Source: Mol Plant Pathol. 2026 Jun 30;27(7):e70307. doi: 10.1111/mpp.70307 (PMC13315810; doi:10.1111/mpp.70307)
Supplement: Supplementary file 1 — Methods S1. Experimental procedures. [file MPP-27-e70307-s009.docx]

**Experimental procedures**

**Plant materials and growth conditions**

Wild-type (WT) *Nicotiana benthamiana* and transgenic *N. benthamiana* lines expressing AtRLP23 or an RLP23/Cf-9 chimera (hereafter referred to as RLP23 and chimera lines, respectively) were grown in a controlled-environment growth chamber at 25 °C and 75% relative humidity under a 16 h light/8 h dark photoperiod.

**Constructs**

The full-length coding sequence (CDS) of *Arabidopsis thaliana* *RLP23* (AT2G32680) was amplified as described previously (Zhao and Cheng, 2023) and inserted into the binary vector pH35GSKm at the KpnI/XhoI sites using the ClonExpress Ultra One Step Cloning Kit (Vazyme, Nanjing, China) to generate 35S::RLP23. To construct RLP23/Cf-9 chimeras, the extracellular domain of RLP23 was fused in frame to Cf-9-derived C-terminal modules from *Solanum lycopersicum* Cf-9 (Jones et al., 1994) using the same ClonExpress strategy, and the resulting fusion fragments were cloned into pH35GS-Km (KpnI/XhoI) to obtain the chimeric expression constructs shown in Fig. 1A. To generate a GFP-tagged version, the coding sequences of RLP23, RLP23/Cf-9^IC^ and RLP23/Cf-9^TM-IC^ were cloned into the pH35GG-Km vector to produce a C-terminal GFP fusion.

Gene fragments of *SOBIR1* and *NRC3* were amplified from *N. benthamiana* cDNA and inserted into the pTRV2 vector using the same ClonExpress cloning approach. Primers used for construct generation are listed in Table S1. All plasmids were confirmed by Sanger sequencing and introduced into *Agrobacterium tumefaciens* GV3101 for transient expression and stable transformation.

**Immunoblot analysis**

For immunoblot analysis of C-terminally GFP-tagged RLP23, RLP23/Cf-9^TM-IC^, and RLP23/Cf-9^IC^, infiltrated *N. benthamiana* leaf tissues were collected at 48 h after agroinfiltration. Total proteins were extracted using the E/Z extraction method. Protein samples were separated by SDS-PAGE and transferred to a nitrocellulose membrane. GFP-tagged proteins were detected using an anti-GFP antibody (GenScript), followed by an alkaline phosphatase-conjugated secondary antibody. Signals were visualized using BCIP/NBT substrate. Coomassie Brilliant Blue staining was used as a loading control.

**Reactive oxygen species burst assay**

Agrobacterium-mediated transient expression in *N. benthamiana* leaves was performed by syringe infiltration using *A. tumefaciens* GV3101 as described previously (Sang and Macho, 2017). Bacterial cultures were adjusted to an OD_600_ of 0.5 and resuspended in liquid MS medium before infiltration into fully expanded leaves of 4–5-week-old plants. After 48 h, 0.5-cm leaf discs were excised and floated in 200 μL sterile water in white 96-well plates overnight in the dark. The water was then replaced with 200 μL of reaction solution containing 1 μM nlp24 peptide (AIMYSWYFPKDSPVTGLGHRHDWE), derived from PpNLP of *Phytophthora parasitica* (GenScript), 200 μM luminol and 20 μg/mL horseradish peroxidase (HRP) (Sigma-Aldrich). Luminescence (relative light units, RLU) was recorded in real time using a GloMax™ 96 microplate reader (Promega) to quantify ROS production. For stable transgenic lines, leaf discs from 4–5-week-old plants were treated with nlp24 at the same final concentration and ROS production was measured as described above. A final concentration of 1 μM nlp24 was used for all ROS burst assays. Each experiment was repeated three times. Statistical significance was assessed by one-way ANOVA followed by Tukey’s multiple comparison test (p < 0.05).

**Hypersensitive response assay**

Hypersensitive response (HR)-associated cell death assays were performed in *N. benthamiana* leaves using the same agroinfiltration procedure described for the ROS assay. At 48 h after agroinfiltration, infiltrated areas were treated with 5 or 10 μM nlp24 or water, and HR symptoms were monitored and photographed at 4–5 days after treatment. For stable transgenic lines, leaves were directly infiltrated with 0.1–10 μM nlp24 or water as a control.

Different nlp24 concentrations were used depending on the purpose of each assay. A concentration of 10 μM was used as a strong treatment condition for construct comparison, VIGS assays, and repeated-treatment experiments in stable transgenic plants. A concentration of 5 μM was used as an intermediate dose to assess cell-death sensitivity under less saturating conditions, including comparison of untagged and GFP-tagged RLP23/Cf-9^TM-IC^ constructs. For threshold assays, a dose series of 0.1–5 μM nlp24 was used to test whether transient NRC3 expression lowered the threshold for cell-death induction.

To evaluate reproducible cell death in stable lines, selected leaf areas were treated either once (1×) or three times consecutively (3×) with 5 or 10 μM nlp24, with each subsequent infiltration performed after the previous infiltrated solution had dried, approximately 1–2 h later. Photographs were taken 7 days after the final treatment. For transient NRC3 expression assays, Agrobacterium cultures carrying NRC3 were infiltrated at OD600 = 0.3, and leaves were treated with nlp24 at 48 h after agroinfiltration; photographs were taken 5 days after treatment. All HR assays were repeated 3–10 times.

**Stable transformation and selection of transgenic lines**

Stable transformation of *N. benthamiana* was performed using an Agrobacterium-mediated leaf-disc method with minor modifications (Dandekar and Fisk, 2004). Regenerated plantlets were rooted, acclimatized, transferred to potting substrate and grown further in a greenhouse. Transgenic T_0_ plants carrying 35S::*RLP23* or 35S::*RLP23/Cf-9^TM-IC^* were verified by genomic PCR, and T_1_ seeds were collected from confirmed lines. T_1_ seeds were germinated on MS medium containing kanamycin (50 mg/L) to select resistant seedlings, which were grown to maturity and selfed to obtain T_2_ seeds. T_2_ seeds were screened on kanamycin, and kanamycin-resistant, non-segregating T_2_ lines were used for subsequent qPCR and functional assays. Transgene transcript levels in T_2_ plants were quantified by qPCR, and nlp24-induced ROS responses were measured to evaluate immune responsiveness.

**RNA extraction and RT–qPCR analysis**

Total RNA was extracted from transgenic *N. benthamiana* and reverse-transcribed into first-strand cDNA using commercial kits (Vazyme, Nanjing, China) according to the manufacturer’s instructions. RT-qPCR was performed using a QuantStudio Real-Time PCR System (Applied Biosystems, Thermo Fisher Scientific) with SYBR Green qPCR Master Mix (Accurate Biotechnology, Hunan, China) under the following cycling conditions: 95°C for 30 s, followed by 40 cycles of 95°C for 5 s, 60°C for 30 s, and 72°C for 30 s. *NbEF1α* was used as the reference gene. Relative transcript levels were calculated using the 2^-ΔΔCt^ method. Statistical significance was assessed by one-way ANOVA followed by Tukey’s multiple comparison test (p < 0.05) using three independent biological replicates. Primers used for expression analysis are listed in Table S1.

**Seedling growth inhibition assay**

Seedling growth inhibition assays were performed using T_2_ seeds of transgenic *N. benthamiana* lines (RLP23 and RLP23/Cf-9^TM-IC^). Surface-sterilized seeds were germinated on solid MS medium for 7 days. The resulting seedlings were then transferred to liquid MS medium supplemented with either 10 μM nlp24 peptide or water (as a mock control). After an additional 7 days of culture, seedling morphology was documented by photography. To quantify growth inhibition, seedlings were blotted dry to remove excess moisture and weighed immediately. Each experiment was repeated three times. Statistical significance was assessed by one-way ANOVA followed by Tukey’s multiple comparison test (p < 0.05).

**Virus-induced gene silencing assay**

Virus-induced gene silencing (VIGS) was performed using the TRV-based vector system (Liu et al., 2002). Agrobacterium tumefaciens GV3101 cultures carrying pTRV1 and the respective pTRV2 constructs (pTRV2::*NbSOBIR1*, pTRV2::*NbNRC3*, or empty vector control) were mixed at a 1:1 ratio (final OD_600_ = 0.5 each) and infiltrated into cotyledons of 3-week-old plants. A separate group of plants was infiltrated with pTRV2::*NbPDS* to serve as a visual indicator for the onset of silencing. Assays were performed approximately three weeks after agroinfiltration when the *PDS*-silenced plants exhibited clear photobleaching phenotypes. Three independent biological replicates (three plants per replicate, yielding six leaves) were used per treatment. Silencing efficiency was validated by RT-qPCR using *NbEF1α* as the internal reference gene. Statistical significance compared with the empty vector control within each indicated genotype was determined using Welch's unpaired t-test (p < 0.05).

***Phytophthora nicotianae* inoculation and pathogen biomass quantification**

*P.* *nicotianae* was kindly provided by Prof. Tingting Dai and Prof. Qin Xiong and maintained on 10% V8 agar at 25°C for 5–7 d. Detached-leaf inoculation was performed with minor modifications to a published protocol (Wang et al., 2022). Briefly, healthy leaves from 4–5-week-old plants were detached and placed on 1.5% water agar to maintain humidity. Mycelial plugs (1 cm diameter) were excised with a sterile cork borer and placed onto the leaf surface, with four inoculation sites per leaf. Disease symptoms were photographed at 3 dpi.

For pathogen biomass quantification, inoculated tissues were sampled at 1 dpi and 3 dpi. Four leaf discs (2 cm diameter) were collected from inoculation sites on each leaf and pooled as one sample. Total genomic DNA (plant–pathogen mixed DNA) was extracted using a genomic DNA kit (Vazyme, Nanjing, China) and diluted to 50 ng/µL. To quantify pathogen growth, qPCR was performed as described above, using *NbEF1α* as the plant internal reference and *P. nicotianae β-tubulin* as the pathogen marker. Relative pathogen biomass was calculated using the 2^-ΔΔCt^ method. Data were obtained from at least three independent biological replicates (with three technical replicates per sample) and analyzed by one-way ANOVA followed by Tukey’s multiple comparison test (p < 0.05).

To examine the relationship between the synthetic nlp24 peptide and the NLP sequence in the *P. nicotianae* isolate used in this study, primers were designed based on the *P. nicotianae* *NPP1* gene sequence (GenBank: AF352031.1) and are listed in Table S1. The full-length coding sequence of the corresponding *NLP* gene was amplified using cDNA prepared from *P. nicotianae*-infected leaf tissue at 36 hpi. RNA extraction and reverse transcription were performed as described above. PCR products were subjected to Sanger sequencing, and the predicted amino acid sequence was compared with the synthetic nlp24 peptide used in this study.

**References**

Dandekar, A.M. and Fisk, H.J. (2004) Plant transformation: *Agrobacterium*-mediated gene transfer. In: Peña, L. (Ed.) *Transgenic Plants: Methods and Protocols*. Totowa, NJ: Humana Press, pp. 35–46.

Jones, D.A., Thomas, C.M., Hammond-Kosack, K.E., Kurti, P.J.B. & Jones, J.D.G. (1994) Isolation of the tomato *Cf-9* gene for resistance to *Cladosporium fulvum* by transposon tagging. *Science*, **266**, 789–793.

Liu, Y., Schiff, M. & Dinesh-Kumar, S.P. (2002) Virus-induced gene silencing in tomato. *The Plant Journal*, **31**, 777–786.

Sang, Y. & Macho, A.P. (2017) Analysis of PAMP-triggered ROS burst in plant immunity. In: Shan, L. and He, P. (Eds.) *Plant Pattern Recognition Receptors. Methods in Molecular Biology*. New York, NY: Springer New York, pp. 143–153.

Wang, W., Zhang, J., Cao, Y., Yang, X., Wang, F., Yang, J., et al. (2022) NtbHLH49, a jasmonate-regulated transcription factor, negatively regulates tobacco responses to *Phytophthora nicotianae*. *Frontiers in Plant Science*, **13**, 1073856.

Zhao, L. & Cheng, Q. (2023) Heterologous expression of *Arabidopsis* pattern recognition receptor RLP23 increases broad-spectrum resistance in poplar to fungal pathogens. *Molecular Plant Pathology*, **24**, 80–86.
