# Supplementary material for: An RLP23/Cf‐9TM‐IC Chimeric Receptor Enhances nlp24‐Triggered Immunity and Resistance to Phytophthora nicotianae in Nicotiana benthamiana
Source: Mol Plant Pathol. 2026 Jun 30;27(7):e70307. doi: 10.1111/mpp.70307 (PMC13315810; doi:10.1111/mpp.70307)
Supplement: Supplementary file 2 — Table S1: Primers used in this study. [file MPP-27-e70307-s001.docx]

**Primers used in this study**

| Primer name | Forward and reverse primers  (5´ to 3´) | Note |
| --- | --- | --- |
| Primers for gene cloning | | |
| *RLP23*-F | ggggactctagaataggtaccATGTCAAAGGCGCTTTTGCA | Coding sequence of *AtRLP23* |
| *RLP23*-R | cctcagctacttaagctcgagCTAACGCTTTCTGCGTTTATTCAG |  |
| *RLP23*^ΔIC^-F | ggggactctagaataggtaccATGTCAAAGGCGCTTTTGCA | Coding sequence of *AtRLP23*^ΔIC^ |
| *RLP23*^ΔIC^-R | gctggatattgagttgaAGCAATAACTTGTGCTATTGCCAA |  |
| *RLP23*^ΔTM-IC^-F | gagaacacgggggactctagtATGTCAAAGGCGCTTTTGCA | Coding sequence of *AtRLP23*^ΔTM-IC^ |
| *RLP23*^ΔTM-IC^-R | ctacttaagctcgagggtaccTCTAGAGTTCAACACTTCTTCTTCTTCTTC |  |
| *Cf-9*^IC^-F | gctTCAACTCAATATCCAGCATGGTTT | Coding sequence of *Cf-9*^IC^ |
| *Cf-9*^IC^-R | cctcagctacttaagctcgagCTAATATCTTTTCTTGTGCTTTTTCATTT |  |
| *Cf-9*^TM-IC^-F | ggggactctagaataggtaccctcgagTGGCAGGGGGTTCTCGTG | Coding sequence of *Cf-9*^TM-IC^ |
| *Cf-9*^TM-IC^-R | ccctcagctacttaagctcgaaCTAATATCTTTTCTTGTGCTTTTTCATTT |  |
| RLP23-F | ggggactctagaataggtaccATGTCAAAGGCGCTTTTGCA | Mutating stop codon of RLP23 |
| RLP23-R | gcccttgctcaccatctcgagACGCTTTCTGCGTTTATTCAGAC |  |
| RLP23/Cf-9^TM-IC^-F | ggggactctagaataggtaccATGTCAAAGGCGCTTTTGCA | Mutating stop codon of RLP23/Cf-9^TM-IC^ |
| RLP23/Cf-9^TM-IC^-R | gcccttgctcaccatctcgagATATCTTTTCTTGTGCTTTTTCATTTTC |  |
| RLP23/Cf-9^IC^-F | ggggactctagaataggtaccATGTCAAAGGCGCTTTTGCA | Mutating stop codon of RLP23/Cf-9^IC^ |
| RLP23/Cf-9^IC^-R | gcccttgctcaccatctcgagATATCTTTTCTTGTGCTTTTTCATTTTC |  |
| *SOBIR1*-VIGS-F | CGCAAGGCCTACTGTGAGCTCCAGAAAGTTTTCCAATGGCAGG | Silencing *Nb*SOBIR1 in N. benthamiana |
| *SOBIR1*-VIGS-R | GGGACATGCCCGGGCCTCGAGAATCTTTATCCACCAGATCATGCTG |  |
| *NRC3*-VIGS-F | cgcaaggcctactgtgagctcATGGCAGATGTAGCAGCAGATG | Silencing *Nb*NRC3 in N. benthamiana |
| *NRC3*-VIGS-R | gggacatgcccgggcctcgagAAACCACTGAGCAAATTTATTTTTATCG |  |
| *NRC3*-F | ggggactctagaataggtaccATGGCAGATGTAGCAGCAGATG | Coding sequence of *NbNRC3* |
| *NRC3*-R | cctcagctacttaagctcgagTTACAATCCGAGATCTGGAGGAA |  |
| Primers for qPCR | | |
| q*NbEF1α*-F | AGCTTTACCTCCCAAGTCATC | Internal control for RT-qPCR |
| q*NbEF1α*-R | AGAACGCCTGTCAATCTTGG |  |
| q*RLP23*^ΔTM-IC^-F | AAGGCGGCTATGGTTATACAGA | qRT-PCR for stable transgenic lines expression analysis |
| q*RLP23*^ΔTM-IC^-R | CATAGACAGGGGAATATGGCCT |  |
| q*P.n.β-tubulin*-F | TAACTGCTGCTTGTATGTTCCG | qPCR for *P. nicotianae* biomass quantification |
| q*P.n.β-tubulin*-R | TGAACTCCATCTCGTCCATACC |  |
| q*SOBIR1*-F | AAAGCCAGCAAATGTCCTTCTT | Validation of *NbSOBIR1* silencing efficiency |
| q*SOBIR1*-R | CTAAACTCATCTCAGGCGTGTG |  |
| q*NRC3*-F | TTTCCTGGCGGTTCTGAAATTT | Validation of *NbNRC3* silencing efficiency |
| q*NRC3*-R | GCTTCATGTCTGCAGAACTCAT |  |
