# Supplementary figures and images for: An RLP23/Cf‐9TM‐IC Chimeric Receptor Enhances nlp24‐Triggered Immunity and Resistance to Phytophthora nicotianae in Nicotiana benthamiana
Source: Mol Plant Pathol. 2026 Jun 30;27(7):e70307. doi: 10.1111/mpp.70307 (PMC13315810; doi:10.1111/mpp.70307)

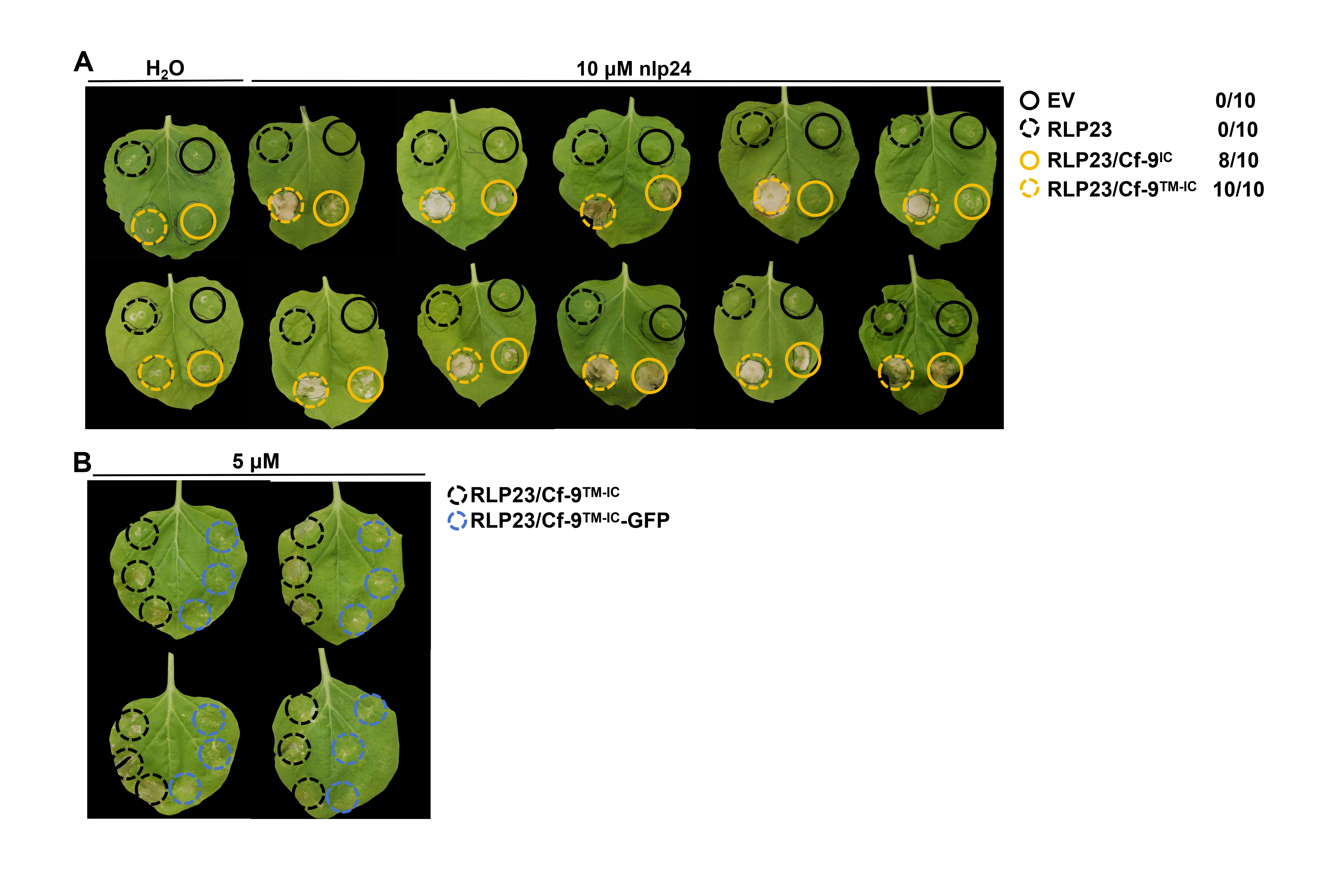

Supplement: Supplementary file 4 — Figure S2: Supplementary cell‐death phenotypes mediated by RLP23/Cf‐9 chimeric receptors in Nicotiana benthamiana. (A) Cell‐death phenotypes induced by the indicated constructs after treatment with H2O or 10 μM nlp24. At 48 h after agroinfiltration, infiltration sites were treated with H2O or nlp24, and photographs were taken 4 days later. The numbers on the right indicate the number of leaves showing macroscopic cell death out of the total number of leaves tested. Leaves were scored as positive when visible dry cell‐death symptoms were observed within the nlp24‐treated infiltration site, including patchy dry areas outside the injection wound; weak chlorosis alone or no visible cell‐death symptoms was not counted as positive. (B) Effect of C‐terminal GFP tagging on RLP23/Cf‐9TM‐IC‐mediated cell death. Leaves expressing untagged RLP23/Cf‐9TM‐IC or C‐terminally GFP‐tagged RLP23/Cf‐9TM‐IC were treated with 5 μM nlp24 at 48 h after agroinfiltration, and photographs were taken 4 days later. [file MPP-27-e70307-s008.tif]

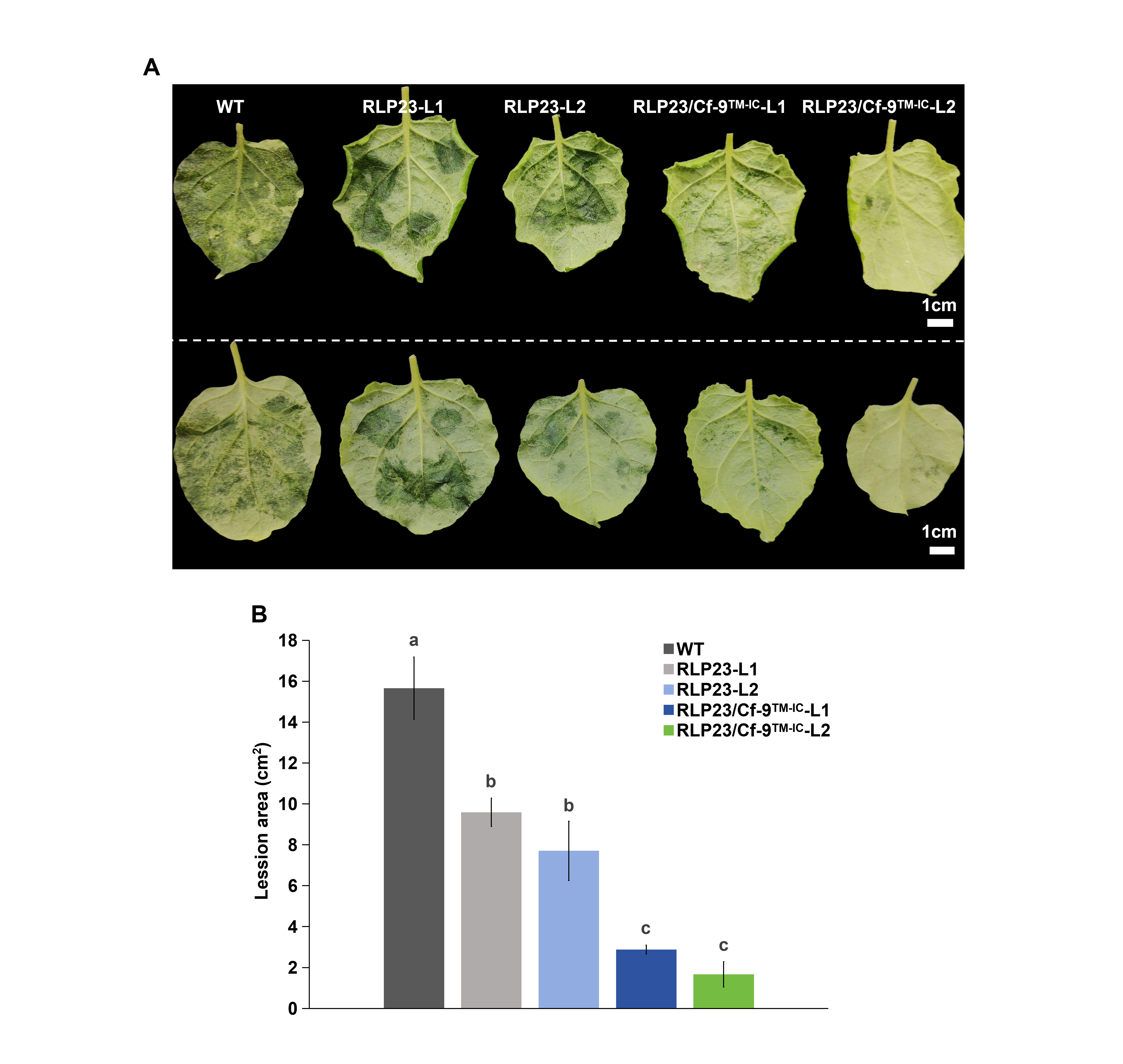

Supplement: Supplementary file 10 — Figure S8: Additional representative phenotypes and lesion area quantification of Phytophthora nicotianae infection on detached leaves. (A) Additional representative detached‐leaf infection phenotypes of wild‐type (WT), RLP23 transgenic lines, and RLP23/Cf‐9TM‐IC transgenic lines at 3 days post‐inoculation (dpi) with P. nicotianae . (B) Quantification of lesion areas in detached leaves at 3 dpi. Lesion areas were measured from photographed leaves using ImageJ software. Data are presented as means ± SEM. Different letters indicate significant differences among genotypes (p < 0.05, one‐way ANOVA followed by Tukey's multiple comparison test). [file MPP-27-e70307-s004.tif]
